# Sleep inequities and associations between poor sleep and mental health for school-aged children: findings from the New Zealand Health Survey

**DOI:** 10.1093/sleepadvances/zpad049

**Published:** 2023-11-18

**Authors:** Diane Muller, T Leigh Signal, Mathangi Shanthakumar, Sarah-Jane Paine

**Affiliations:** Sleep/Wake Research Centre, School of Health Sciences, College of Health, Massey University, Wellington, New Zealand; Sleep/Wake Research Centre, School of Health Sciences, College of Health, Massey University, Wellington, New Zealand; Environmental Health Intelligence NZ, Massey University, Wellington, New Zealand; Te Kupenga Hauora Māori, Faculty of Medical and Health Sciences, University of Auckland, Auckland, New Zealand

**Keywords:** anxiety, depression, Indigenous sleep, race/ethnicity, sleep disparities, sleep health, snoring, sleep disorders, socioeconomic position

## Abstract

In Aotearoa/New Zealand, ethnic inequities in sleep health exist for young children and adults and are largely explained by inequities in socioeconomic deprivation. Poor sleep is related to poor mental health for these age groups but whether sleep inequities and associations with mental health exist for school-aged children is unclear. We aimed to (1) determine the prevalence of poor sleep health including sleep problems by ethnicity, (2) examine social determinants of health associated with poor sleep, and (3) investigate relationships between poor sleep and mental health for 5–14-year-olds using cross-sectional New Zealand Health Survey data (*n* = 8895). Analyses included weighted prevalence estimates and multivariable logistic regression. Short sleep was more prevalent for Indigenous Māori (17.6%), Pacific (24.5%), and Asian (18.4%) children, and snoring/noisy breathing during sleep was more prevalent for Māori (29.4%) and Pacific (28.0%) children, compared to European/Other (short sleep 10.2%, snoring/noisy breathing 17.6%). Ethnicity and neighborhood socioeconomic deprivation were independently associated with short sleep and snoring/noisy breathing during sleep. Short sleep was associated with increased odds of anxiety, attention deficit hyperactivity disorder, and activity-limiting emotional and psychological conditions after adjusting for ethnicity, deprivation, age, and gender. In addition, long sleep was independently associated with increased odds of depression. These findings demonstrate that for school-aged children ethnic inequities in sleep exist, socioeconomic deprivation is associated with poor sleep, and poor sleep is associated with poor mental health. Sociopolitical action is imperative to tackle social inequities to support sleep equity and mental health across the lifecourse.

**Statement of Significance**

We provide evidence that unjust sleep inequities exist for Indigenous Māori and minoritized (Pacific and Asian) school-aged children in Aotearoa/New Zealand. Stark inequities in socioeconomic deprivation for Māori and Pacific children and, to a lesser degree, Asian children partially account for these sleep inequities. Short and long sleep are also independently associated with poor mental health for school-aged children in Aotearoa/New Zealand. Considered in conjunction with previous research, results suggest that sleep inequities start early in life, span the majority if not all of the lifecourse, and have significant implications for mental well-being. Research is required to investigate additional social determinants of school-aged children’s sleep, such as racism. Findings can inform equity-based policies and sociopolitical action to achieve sleep equity.

## Introduction

A growing body of evidence indicates that racial/ethnic sleep inequities exist in childhood. For example, in the United States, a disproportionate number of black and Hispanic school-aged children and adolescents have shorter or poorer quality sleep than white youth [1, 2]. Child sleep health inequities also exist in Aotearoa/New Zealand (NZ), with Pacific and Asian preschoolers disproportionately impacted by short sleep compared with European/Other preschoolers [3], and Indigenous Māori preschoolers more likely to experience short sleep, inconsistent sleep timing between weekdays and weekends and disturbed sleep than non-Māori preschoolers [4–6].

Unfair and unjust sleep health inequities between Indigenous and non-Indigenous peoples must be considered within the broader context of colonialism and racism [7] which are global systems of oppression that operate to structure access to power and privilege in society based on beliefs in racial hierarchies. For example, Māori are over-represented in areas with the greatest socioeconomic deprivation, which is a consequence of decades of institutional racism stemming from colonization [8]. Prior research has shown that neighborhood socioeconomic deprivation [9] is a significant determinant of both preschooler and adult poor sleep health and accounts for some, but not all, of the inequities in sleep between Māori and non-Māori [6]. Furthermore, experiences of racial discrimination have also been identified as contributing to ethnic inequities in sleep disturbances between Māori and non-Māori adults [10].

An important research agenda is to investigate how structural determinants may limit opportunities for good sleep health for other groups that experience marginalization, including by ethnicity and age, who have received less research attention to date. Doing so is vital for informing policy and guiding health practices that support sleep equity for groups disadvantaged by systemic social inequities. In NZ, under-researched groups in relation to sleep include school-aged Indigenous Māori and minoritized Pacific and Asian children. Examining relationships between sleep inequities and well-being has also been identified as a research priority [1]. Concerningly, associations have been identified between poor sleep health and poor mental health for preschool-aged children [11] and adults [12] in NZ; however, these relationships are less well understood for school-aged children.

Therefore, three key research gaps warrant investigation. Namely, the identification of whether sleep inequities exist for school-aged Māori, Pacific, and Asian children; the examination of relationships between structural determinants and sleep inequities in school-aged children; and the investigation of associations between school-aged children’s sleep and mental health in NZ. Our primary research aims were to (1) determine the prevalence of poor sleep health including sleep problems by ethnicity, (2) examine social determinants of health associated with poor sleep, and (3) investigate relationships between poor sleep and mental health, using data from a large nationally representative sample of 5- to 14-year-olds collected as part of the New Zealand Health Survey (NZHS) [13].

## Methods

### Datasets and participants

Ethics approval to access NZHS data and undertake this research was granted by the Massey University Human Ethics Southern A Committee (SOA 21/13).

The NZHS continuously monitors population health in NZ and provides evidence to inform health policy. It uses a multi-stage, stratified, probability-to-size sampling design plus a dual-frame approach to achieve increased sampling of Māori, Pacific, and Asian ethnic groups [13]. Calibrated weights are provided with data to enable nationally representative estimates to be calculated for health indicators. Surveys are administered to one eligible adult (≥15 years) and up to one child (0–14 years) per selected residence using face-to-face and computer-assisted data collection methods. For children, this is by proxy via their primary caregiver. Surveys are cross-sectional in nature and are administered to different households from 1 year to the next. They comprise a set of core health and sociodemographic questions plus additional modules on different health-related topics that change annually. NZHS data were provided to the research team as Confidentialized Unit Record Files.

As our focus was school-aged children, NZHS child datasets were filtered to only include 5- to 14-year-olds, to reflect the fact that most children in New Zealand start school at 5 years of age and that those aged 15 years or over are considered an “adult” in the NZHS. This study analyzed cross-sectional data collected from independent samples in 2013–2014, 2017–2018, and 2018–2019, with these years selected as they were the instances when child sleep data were available.

### Sociodemographic measures

Several sociodemographic variables relevant to the current study were measured in the surveys using Statistics New Zealand standardized questions for household social surveys in NZ [13]. The social construct of child *ethnicity* (“Which ethnic group or groups does [child] belong to?”) was categorized in two ways in datasets, in line with standardized procedures for collecting and reporting ethnicity data in the health and disability sector in NZ [14]:

Firstly, total response Māori, Pacific, and Asian whereby each child was reported in each of these group/s that they belonged to i.e. if a child identified as Māori and Pacific they were allocated to both groups. It is important to note that “Pacific” and “Asian” are umbrella groupings incorporating multiple ethnicities from Pacific and Asian regions respectively, such as children whose ethnic identity included Tongan or Samoan (“Pacific”) or Chinese or Indian (“Asian”). The total response classification system has the advantage of maximizing responses for numerically smaller population groups, such as Pacific peoples, in NZ. However, the total response approach creates ethnic groups that are not mutually exclusive which limits the ability to compare prevalence estimates by ethnicity given a high degree of overlap. Therefore, we also used a sole European/Other group as the comparator group in this analysis to reflect the position of privilege held by the dominant European ethnic grouping stemming from colonization in NZ [7]. The European/Other variable included children only identifying as belonging to a European (e.g. New Zealand European) or “other” (e.g. Middle Eastern, Latin American, or African) grouping. While it would have been ideal to disaggregate the European and “other” groupings, this was not possible due to the format of this variable provided by the 2013–2014, 2017–2018, and 2018–2019 NZHS [15]. However, in an earlier instance of the NZHS (2006–2007), this was predominantly comprised of European ethnic groups alongside a small proportion (approximately 1%) of people who identified their ethnicity in the “other” category [16].

Secondly, prioritized output whereby each child was allocated to a single ethnic group in prioritized order of Māori, Pacific, Asian, and European/Other [14].

Child *gender* was categorized as male/female and child *age* as 5–9/10–14 years. *Neighborhood deprivation* was measured using NZDep2013 [9] which is a small area-level, composite measure of relative socioeconomic deprivation based on eight dimensions (communication, income, employment, educational qualifications, home ownership, support, living space, and living conditions) measured in the New Zealand Census (quintile 1 = least deprived, quintile 5 = most deprived) widely used in NZ as an indicator of socioeconomic position [17]. In addition, the 2013–2014 NZHS module included a measure of *individual-level deprivation* the New Zealand Index of Socioeconomic Deprivation for Individuals (NZiDep), completed by the child’s primary caregiver [17]. NZiDep scores range from 1 (no deprivation characteristics; least deprived) to 5 (5 or more deprivation characteristics; most deprived), based on questions about an individual’s experience/s of deprivation in the previous 12 months such as being out of paid work and feeling cold due to saving money on heating.

### Sleep measures

The available NZHS sleep health indicator was child sleep duration, which was measured using the question “How many hours of sleep does [child] usually get in a 24-hour period, including all naps and sleeps?” We prepared a categorical *sleep duration* variable to indicate if children had durations within or outside of the recommended range for their age i.e. short (5–13 years: <9 hours; 14 years: <8 hours), recommended (5–13 years: 9–11 hours; 14 years: 8–10 hours), or long (5–13 years: >11 hours; 14 years: >10 hours). This was based on NZ sleep duration guidelines [18] which are informed by US National Sleep Foundation guidelines [19] with the exception that “school-aged” children are classified as 5–13 years in NZ versus 6–13 years in the United States.

From the 2017–2018 survey onwards the sleep duration question was part of the suite of core NZHS child health questions, but it was not included in the years 2014–2015, 2015–2016, or 2016–2017. The 2013–2014 NZHS included a child sleep module which, in addition to the sleep duration question, incorporated questions on sleep problems, namely *snoring and/or noisy breathing during sleep* (“In the last 4 weeks did [child] snore or breathe noisily on most nights, whilst sleeping?” yes/no) and diagnosed *sleep disorders* (“Have you ever been told by a doctor or other health professional that [child] has a sleep disorder?” yes/no).

### Mental health measures

Mental health measures included questions on diagnosis (“Have you ever been told by a doctor that [child] has . . .?”) *depression* (yes/no)*, anxiety* (yes/no), and attention deficit disorder/attention deficit hyperactivity disorder (*ADD/ADHD*) (yes/no), which were included in all three surveys. Additional mental health variables only available in the 2013–2014 NZHS were *activity-limiting emotional problem* (“Most children have occasional emotional, nervous, or behavioral problems. Does [child] have any of these problems long term, that limits the type or amount of activity that [child] can do?” yes/no), and *activity-limiting psychological condition* (“Does a long-term psychological or mental health condition make it difficult for [child] to do everyday activities?” yes/no).

### Statistical analysis

Statistical analyses were conducted using SAS software, Version 9.4 (SAS Institute Inc., Cary, NC, USA.). Demographic characteristics of 5- to 14-year-old children in the 2013–2014, 2017–2018, and 2018–2019 samples were described using percentages and 95% confidence intervals (95% CI) stratified by ethnicity (total response Māori, Pacific, Asian, and sole European/Other as the comparator group). Weighted prevalence estimates and 95% CI were produced for categorical sleep variables, stratified by ethnicity (total response Māori, Pacific, Asian, and sole European/Other) to indicate where ethnic inequities exist in child sleep.

Unadjusted and adjusted logistic regression models were run to investigate associations between child ethnicity and poor sleep and the contribution of socioeconomic deprivation to these relationships, controlling for gender and age. Therefore, the dependent variable was sleep (one sleep variable per model) and the independent variable in unadjusted models was ethnicity (prioritized ethnicity, with “European/Other” as the reference group). Independent variables in adjusted models were child ethnicity, neighborhood deprivation (NZDep2013, reference = quintile 1), individual-level deprivation (for analyses of 2013–2014 NZHS data; NZiDep scores, reference = score of 1), age (reference = 5–9-years) and gender (reference = male). Unadjusted and adjusted logistic regression models were similarly structured to examine associations between poor sleep (independent variable, one per model) and mental health (dependent variable, one per model). Adjusted models included independent variables of sleep, child ethnicity, neighborhood deprivation, individual-level deprivation (for analyses using the 2013–2014 NZHS only), age, and gender.

## Results

In this sample of 5- to 14-year-olds (combined sample from 2013 to 2014, 2017 to 2018, and 2018 to 2019 NZHS: *n* = 8895), approximately one-half of Māori, two-thirds of Pacific and one-quarter of Asian children lived in areas with the greatest socioeconomic deprivation compared to just over one-tenth of European/Other children (Table 1). In addition, a significantly larger proportion of caregivers of Māori and Pacific children reported multiple individual experiences of deprivation (NZiDep scores of 4 or 5) in the past year compared to caregivers of European/Other children.

**Table 1.** Description of Demographic Characteristics of 5- to 14-Year-Old Children in the Combined Sample From 2013 to 2014 (*n* = 2912), 2017 to 2018 (*n* = 3063), and 2018 to 2019 (2920) New Zealand Health Surveys, Stratified by Child Ethnicity^a^

	Māori (*n* = 3294)	Pacific (*n* = 1313)	Asian (*n* = 1084)	European/Other (*n* = 3774)
	% (95% CI)	% (95% CI)	% (95% CI)	% (95% CI)
Age				
5–9 years	49.5 (47.7 to 51.2)	51.5 (48.8 to 54.2)	51.0 (48.0 to 54.0)	48.5 (46.9 to 50.1)
10–14 years	50.5 (48.8 to 52.3)	48.5 (45.8 to 51.2)	49.0 (46.0 to 52.0)	51.5 (49.9 to 53.1)
Gender				
Female	49.0 (47.3 to 50.7)	49.1 (46.4 to 51.8)	49.3 (46.3 to 52.2)	48.6 (47.0 to 50.2)
Male	51.0 (49.3 to 52.7)	50.9 (48.2 to 53.6)	50.7 (47.8 to 53.7)	51.4 (49.8 to 53.0)
Neighborhood deprivation^b^				
1 (least deprived)	7.3 (6.5 to 8.2)	4.2 (3.2 to 5.4)	15.0 (12.9 to 17.2)	22.1 (20.7 to 23.4)
2	9.1 (8.2 to 10.1)	6.5 (5.2 to 7.9)	18.0 (15.7 to 20.3)	21.9 (20.6 to 23.2)
3	14.1 (12.9 to 15.2)	10.1 (8.4 to 11.7)	18.9 (16.6 to 21.2)	20.9 (19.6 to 22.2)
4	22.9 (21.4 to 24.3)	19.3 (17.1 to 21.4)	24.4 (21.8 to 26.9)	21.4 (20.1 to 22.7)
5 (most deprived)	46.6 (44.9 to 48.3)	59.9 (57.3 to 62.6)	23.7 (21.2 to 26.2)	13.7 (12.6 to 14.8)
Individual-level deprivation^c^^,^^d^				
1 (least deprived)	25.7 (23.1 to 28.3)	21.5 (17.5 to 25.5)	52.0 (46.3 to 57.6)	48.3 (45.5 to 51.1)
2	17.8 (15.6 to 20.1)	18.3 (14.6 to 22.1)	17.3 (13.1 to 21.6)	20.1 (17.9 to 22.3)
3	19.0 (16.7 to 21.3)	20.3 (16.4 to 24.2)	15.7 (11.6 to 19.8)	15.8 (13.8 to 17.9)
4	21.2 (18.8 to 23.6)	22.5 (18.4 to 26.6)	12.4 (8.7 to 16.1)	9.3 (7.7 to 10.9)
5 (most deprived)	16.3 (14.1 to 18.5)	17.4 (13.7 to 21.0)	2.6 (1.1 to 5.1)	6.5 (5.1 to 7.8)

CI = confidence interval.

^a^Reported by total Māori, total Pacific, total Asian and sole European/Other child ethnicity (NB: European/Other grouping was based on the format of the data made available to the team by Statistics New Zealand);

^b^Area-level New Zealand Deprivation Index 2013 (NZDep2013) quintiles indicative of relative socioeconomic deprivation based on eight dimensions of deprivation (communication, income, employment, qualifications, home ownership, support, living space, and living conditions) measured in the New Zealand Census;

^c^Only available in the 2013–2014 New Zealand Health Survey;

^d^Based on New Zealand Index of Socioeconomic Deprivation for Individuals (NZiDep) scores: 1 = no deprivation characteristics, 2 = 1 deprivation characteristic, 3 = 2 deprivation characteristics, 4 = 3 or 4 deprivation characteristics, 5 = ≥5 deprivation characteristics, based on responses to questions about experiences of deprivation in the past 12 months (buying cheaper food to pay for other things needed; being out of paid work for more than 1 month; being on a means-tested benefit; feeling cold to save on heating costs; making use of special food grants or food banks; wearing worn-out shoes with holes because cannot afford replacement; going without fresh fruit and vegetables often to pay for other things needed; help from community organizations in the form of clothes or money.

**Figure 1.**
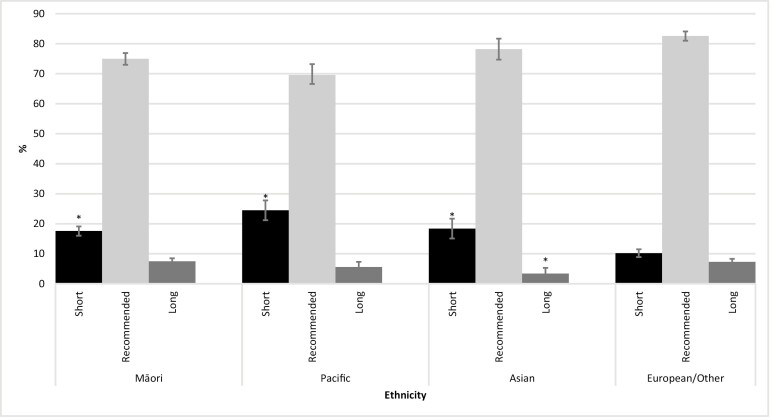
Weighted prevalence estimates of short, recommended, and long sleep durations, by total Māori, Pacific, Asian, and sole European/Other ethnicity. Note. Sleep durations categorized as short (5–13 years: <9 hours; 14 years: <8 hours), recommended (5–13 years: 9–11 hours; 14 years: 8–10 hours), or long (5–13 years: >11 hours; 14 years: >10 hours); * Denotes statistically significant difference in prevalence compared to European/Other.

A significantly larger proportion (% [95% CI]) of Māori (17.6 [16.0 to 19.1]), Pacific (24.5 [21.2 to 27.8]), and Asian (18.4 [15.1 to 21.7]) children had short sleep durations and a significantly smaller proportion of Asian children had long sleep durations (3.4 [2.0 to 5.3]), compared to European/Other (short: 10.2 [8.9 to 11.5], long: 7.3 [6.3 to 8.3]) children (Figure 1). Additionally (Supplementary Figure S1), a significantly larger proportion of Māori (29.4 [25.9 to 33.0]) and Pacific (28.0 [22.3 to 33.7]) children were reported by caregivers to snore and/or breathe noisily during sleep compared to European/Other (17.6 [14.5 to 20.6]) children. A very small proportion of children had been diagnosed with a sleep disorder by a health professional and, while not statistically significantly different, the prevalence was approximately double for European/Other children (2.5 [1.3 to 4.6]) than for Māori (1.2 [0.4 to 2.7]), and Pacific (1.2 [0.4 to 2.8]) children.

Several unadjusted associations were identified between ethnicity and sleep health (Table 2). Māori and Asian children had approximately twice the odds and Pacific children had approximately three times the odds of having short sleep durations compared to European/Other children. Asian children were also significantly less likely to have long sleep. In addition, Māori and Pacific children had approximately twice the odds of snoring and/or noisy breathing during sleep than European/Other children.

**Table 2.** Results of Unadjusted and Adjusted Logistic Regression Models Investigating Associations Between Ethnicity and Sleep Duration and Snoring and/or Noisy Breathing During Sleep

	Short and long sleep duration^a^		Snoring and/or noisy breathing during sleep^b^^,^^c^
	Short	Long	
	OR (95% CI)	OR (95% CI)	OR (95% CI)
Unadjusted model			
Ethnicity^d^			
Māori	**1.90 (1.61 to 2.24)**	1.13 (0.92 to 1.38)	**1.96 (1.53 to 2.51)**
Pacific	**3.02 (2.29 to 3.99)**	0.81 (0.51 to 1.29)	**1.83 (1.23 to 2.73)**
Asian	**1.87 (1.42 to 2.45)**	**0.46 (0.27 to 0.80)**	1.50 (0.99 to 2.25)
European/Other	Ref	Ref	Ref
Adjusted model^e^			
Ethnicity^d^			
Māori	**1.52 (1.29 to 1.78)**	1.02 (0.81 to 1.28)	**1.59 (1.23 to 2.06)**
Pacific	**2.30 (1.73 to 3.07)**	0.70 (0.42 to 1.18)	1.45 (0.96 to 2.19)
Asian	**1.76 (1.35 to 2.31)**	**0.44 (0.25 to 0.76)**	1.38 (0.93 to 2.07)
European/Other	Ref	Ref	Ref
Age			
5–9	Ref	Ref	Ref
10–14	**1.96 (1.68 to 2.28)**	**0.29 (0.22 to 0.38)**	0.88 (0.70 to 1.11)
Gender			
Female	0.93 (0.80 to 1.08)	1.09 (0.88 to 1.34)	**0.64 (0.50 to 0.83)**
Male	Ref	Ref	Ref
Neighborhood deprivation^f^			
1 (least deprived)	Ref	Ref	Ref
2	1.04 (0.72 to 1.50)	0.92 (0.61 to 1.40)	1.55 (0.95 to 2.54)
3	1.20 (0.82 to 1.77)	1.03 (0.70 to 1.53)	1.44 (0.88 to 2.35)
4	**1.54 (1.08 to 2.21)**	1.17 (0.78 to 1.74)	1.45 (0.90 to 2.34)
5 (most deprived)	**1.98 (1.46 to 2.69)**	1.15 (0.76 to 1.75)	**1.76 (1.11 to 2.80)**
Individual-level deprivation^c^^,^^g^	—	—	
1 (least deprived)			Ref
2			1.04 (0.73 to 1.48)
3			1.16 (0.85 to 1.59)
4			1.37 (0.92 to 2.03)
5 (most deprived)			1.18 (0.75 to 1.86)

Sampling weights were applied to all logistic regression models. Bold signifies statistical significance.

OR = odds ratio; CI = confidence interval; Ref = reference category.

^a^Reference = “recommended” sleep duration (5–13 years: 9–11 hours; 14 years: 8–10 hours);

^b^Reference = no snoring/noisy breathing during sleep;

^c^Only available in the 2013–2014 New Zealand Health Survey;

^d^Ethnicity in prioritized order of Māori, Pacific, Asian, European/Other (NB: European/Other grouping was based on the format of the data made available to the team by Statistics New Zealand);

^e^Concurrently adjusted for ethnicity, age, gender, neighborhood deprivation (plus individual-level deprivation for the snoring/noisy breathing during sleep model);

^f^Area-level New Zealand Deprivation Index 2013 (NZDep2013) quintiles indicative of relative socioeconomic deprivation based on eight dimensions of deprivation (communication, income, employment, qualifications, home ownership, support, living space, and living conditions);

^g^Based on New Zealand Index of Socioeconomic Deprivation for Individuals (NZiDep) scores: 1 = no deprivation characteristics, 2 = 1 deprivation characteristic, 3 = 2 deprivation characteristics, 4 = 3 or 4 deprivation characteristics, 5 = ≥5 deprivation characteristics, based on responses to questions about experiences of deprivation in the past 12 months (buying cheaper food to pay for other things needed; being out of paid work for more than one month; being on a means-tested benefit; feeling cold to save on heating costs; making use of special food grants or food banks; wearing worn-out shoes with holes because cannot afford replacement; going without fresh fruit and vegetables often to pay for other things needed; help from community organizations in the form of clothes or money).

Adjusting for socioeconomic deprivation, age, and gender (adjusted models, Table 2) partially attenuated these relationships but did not fully account for associations between ethnicity and sleep. A dose–response relationship was observed between neighborhood deprivation and sleep, with increasing neighborhood socioeconomic deprivation associated with increased odds of short sleep. Living in neighborhoods with the greatest deprivation was also associated with almost twice the odds of snoring and/or breathing noisily during sleep, compared to living in least deprived areas. Due to only a small number of children having a diagnosed sleep disorder (Figure 1), regression models examining associations between ethnicity and sleep disorders could not be reliably interpreted and are therefore not presented.

Univariate associations were identified between sleep duration and mental health, whereby short sleep was associated with higher odds of diagnosed depression, anxiety, and ADD/ADHD (unadjusted models, Table 3). Short sleep remained a significant predictor of diagnosed anxiety and ADD/ADHD, after concurrently adjusting for ethnicity, neighborhood deprivation, age, and gender (adjusted models, Table 3). In addition, long sleep was independently associated with nearly three times higher odds of diagnosed depression (adjusted model, Table 3).

**Table 3.** Unadjusted and Adjusted Logistic Regression Models (With Weighting): Associations Between Short and Long Sleep Durations and Poor Mental Health

	Depression^a^^,^^b^	Anxiety^a^^,^^b^	ADD/ADHD^a^^,^^b^	Activity-limiting emotional problem^c^^,^^d^	Activity-limiting psychological condition^d^^,^^e^
	OR (95% CI)	OR (95% CI)	OR (95% CI)	OR (95% CI)	OR (95% CI)
Unadjusted model					
Sleep duration^f^					
Short	**2.13 (1.12 to 4.07)**	**1.43 (1.00 to 2.05)**	**1.90 (1.19 to 3.04)**	**1.80 (1.20 to 2.71)**	**2.82 (1.51 to 5.25)**
Recommended	Ref	Ref	Ref	Ref	Ref
Long	1.66 (0.64 to 4.30)	1.34 (0.82 to 2.18)	0.79 (0.39 to 1.59)	0.71 (0.38 to 1.31)	1.28 (0.55 to 2.94)
Adjusted model^g^					
Sleep duration^f^					
Short	1.63 (0.85 to 3.13)	**1.53 (1.05 to 2.22)**	**1.84 (1.17 to 2.89)**	**1.68 (1.11 to 2.55)**	**2.67 (1.35 to 5.25)**
Recommended	Ref	Ref	Ref	Ref	Ref
Long	**2.78 (1.07 to 7.22)**	1.56 (0.95 to 2.55)	0.92 (0.45 to 1.89)	0.66 (0.35 to 1.24)	1.35 (0.57 to 3.20)
Ethnicity^h^					
Māori	1.86 (0.92 to 3.78)	0.86 (0.62 to 1.18)	0.98 (0.61 to 1.56)	0.99 (0.62 to 1.59)	1.63 (0.76 to 3.48)
Pacific	0.42 (0.06 to 2.94)	**0.08 (0.03 to 0.21)**	**0.14 (0.03 to 0.58)**	0.49 (0.23 to 1.02)	0.44 (0.15 to 1.28)
Asian	0.21 (0.03 to 1.54)	**0.19 (0.07 to 0.53)**	**0.19 (0.06 to 0.61)**	0.62 (0.28 to 1.38)	1.17 (0.40 to 3.40)
European/Other	Ref	Ref	Ref	Ref	Ref
Age					
5–9	Ref	Ref	Ref	Ref	Ref
10–14	**9.34 (3.58 to 24.35)**	**1.92 (1.47 to 2.51)**	**1.90 (1.33 to 2.69)**	1.22 (0.81 to 1.83)	1.48 (0.81 to 2.71)
Gender					
Female	**0.56 (0.32 to 0.96)**	**0.67 (0.50 to 0.89)**	**0.22 (0.14 to 0.34)**	**0.41 (0.26 to 0.65)**	**0.31 (0.18 to 0.53)**
Male	Ref	Ref	Ref	Ref	Ref
Neighborhood deprivation^i^					
1 (least deprived)	Ref	Ref	Ref	Ref	Ref
2	1.24 (0.26 to 5.99)	1.67 (0.98 to 2.82)	**3.03 (1.43 to 6.42)**	2.18 (0.91 to 5.18)	1.87 (0.51 to 6.81)
3	1.02 (0.16 to 6.29)	1.08 (0.65 to 1.82)	1.78 (0.80 to 3.98)	1.50 (0.69 to 3.24)	1.99 (0.65 to 6.10)
4	1.84 (0.44 to 7.68)	1.53 (0.99 to 2.35)	**2.40 (1.09 to 5.28)**	1.65 (0.77 to 3.57)	1.96 (0.67 to 5.75)
5 (most deprived)	1.50 (0.36 to 6.22)	1.10 (0.66 to 1.83)	**2.46 (1.20 to 5.06)**	1.64 (0.67 to 3.98)	1.17 (0.32 to 4.25)
Individual-level deprivation^d^^,^^j^	—	—	—		
1 (least deprived)				Ref	Ref
2				1.16 (0.65 to 2.05)	1.99 (0.81 to 4.89)
3				1.36 (0.78 to 2.34)	1.36 (0.48 to 3.85)
4				**1.83 (1.07 to 3.12)**	2.53 (0.93 to 6.86)
5 (most deprived)				**3.17 (1.87 to 5.40)**	1.84 (0.61 to 5.53)

OR = odds ratio; CI = confidence interval; Ref = reference category. Bold signifies statistical significance.

^a^Diagnosed by a health professional;

^b^Included in the 2013–2014, 2017–2018, and 2018–2019 New Zealand Health Survey;

^c^Based on response to the question “Most children have occasional emotional, nervous, or behavioral problems. Does [child] have any of these problems long-term, that limits the type or amount of activity that [he/she] can do?”;

^d^Only included in the 2013–2014 New Zealand Health Survey;

^e^Based on response to the question “Does a long-term psychological or mental health condition make it difficult for [child] to do everyday activities?”;

^f^Categorized as short (5–13 years: <9 hours; 14 years: <8 hours), recommended (5–13 years: 9–11 hours; 14 years: 8–10 hours), or long (5–13 years: >11 hours; 14 years: >10 hours) duration;

^g^Concurrently adjusted for sleep, ethnicity, age, gender, and neighborhood deprivation plus individual-level deprivation for emotional and mental health problem models;

^h^Ethnicity in prioritized order of Māori, Pacific, Asian, European/Other (NB: European/Other grouping was based on the format of the data made available to the team by Statistics New Zealand);

^i^Area-level New Zealand Deprivation Index 2013 (NZDep2013) quintiles indicative of relative socioeconomic deprivation based on eight dimensions of deprivation (communication, income, employment, qualifications, home ownership, support, living space, and living conditions);

^j^Based on New Zealand Index of Socioeconomic Deprivation for Individuals (NZiDep) scores: 1 = no deprivation characteristics, 2 = 1 deprivation characteristic, 3 = 2 deprivation characteristics, 4 = 3 or 4 deprivation characteristics, 5 = ≥5 deprivation characteristics, based on responses to questions about experiences of deprivation in the past 12 months (buying cheaper food to pay for other things needed; being out of paid work for more than 1 month; being on a means-tested benefit; feeling cold to save on heating costs; making use of special food grants or food banks; wearing worn-out shoes with holes because cannot afford replacement; going without fresh fruit and vegetables often to pay for other things needed; help from community organizations in the form of clothes or money.

Results of analyses of 2013–2014 NZHS data indicated that short sleep was also associated with almost twice the odds of activity-limiting emotional problems and almost three times greater odds of activity-limiting psychological conditions, with or without adjusting for ethnicity, neighborhood- and individual-­level deprivation, age and gender (unadjusted and adjusted models, Table 3). Unadjusted associations were also identified between snoring and/or noisy breathing during sleep and increased odds of diagnosed ADD/ADHD, and activity-limiting emotional or psychological problems (unadjusted models, Supplementary Table S1). However, these associations were no longer significant after adjusting for ethnicity, neighborhood- and individual-level deprivation, age, and gender (adjusted models, Supplementary Table S1). Due to the small proportion of children with diagnosed sleep disorders, associations with mental health variables could not be reliably calculated.

## Discussion

To the best of our knowledge, this is the first study to investigate sleep inequities and relationships between sleep and mental health for 5- to 14-year-olds in NZ using large, nationally representative survey data and a social determinants of health lens. We found that ethnic inequities exist in school-aged children’s sleep and are partially explained by socioeconomic deprivation, reflecting patterns observed in early childhood [3–6] and adulthood [6]. We established independent relationships between short sleep and poor mental health in school-aged children, which were also similar to those demonstrated in young children [11] and adults [12]. Independent associations were also found between long sleep and depression, thus adding to an under-­studied area of research [20]. Considered in conjunction with previous research, findings indicate that sleep inequities start early in life and span the majority, if not all, of the lifecourse in NZ which has significant implications for mental health.

Māori children in our study were disproportionately impacted by short sleep durations compared to European/Other children. In addition, a greater proportion of Māori children were reported by their caregivers to snore or breathe noisily during sleep on a regular basis which is indicative of potential sleep-disordered breathing (SDB) [21]. This parallels a higher prevalence of short sleep for Māori 2-year-olds compared to European/Other [3] and Māori 3–4-year-olds compared to non-Māori [4], and a higher prevalence of short sleep [22] and SDB [23] for Māori adults compared to non-Māori.

Thus, we add to a comprehensive body of NZ research that indicates that unjust sleep inequities impact Indigenous peoples across multiple life stages. Our findings also compare with a small but growing body of Australian research demonstrating that Indigenous children are disproportionately impacted by short sleep and symptoms of SDB compared to non-I ndigenous children [24, 25]. The fact that Indigenous children are at greater risk of not being afforded the basic human right of healthy sleep is in breach of the United Nations Declaration on the Rights of Indigenous Peoples [26] and warrants urgent action.

Highlighting structural determinants associated with Indigenous sleep inequities, almost half (47%) of Māori children in our study lived in neighborhoods with the greatest socioeconomic deprivation and over one-third (38%) lived with a caregiver who reported multiple experiences of deprivation in the previous year, as indicated by NZiDep scores of 4 or 5 [27], compared to 14% and 16% respectively of European/Other children. The fact that such a large and significantly greater proportion of Māori children were living in material hardship compared to the predominant ethnic grouping of European/Other children is reflective of institutional racism [28] and the stark and entrenched social inequities experienced by Māori [8] stemming from colonization [7]. This was a key factor associated with sleep inequities experienced by Māori school-aged children in our study, as it is for Māori preschoolers and adults [6].

We also contribute to the understanding of sleep inequities experienced by minoritized children worldwide and provide further evidence for the importance of tackling their root causes. We add to the literature by providing evidence that sleep inequities also exist for Pacific school-aged children in NZ. Approximately one quarter (24.5%) of Pacific children were estimated to have short sleep durations and one-third (28.0%) to snore or breathe noisily during sleep, which was significantly greater than for European/Other children. This builds on inequitable patterns of sleep health reported for Pacific preschoolers [3], adolescents [29], and adults [30] and suggests that sleep inequities impact Pacific peoples across the lifecourse in NZ. Pacific children in our study were profoundly over-represented in areas with high socioeconomic deprivation, with approximately two-thirds living in the most socioeconomically deprived neighborhoods. Approximately 40% of Pacific children also lived in deprived households, as indicated by their caregiver reporting multiple experiences of socioeconomic deprivation (NZiDep scores of 4 or 5) in the past year. This reflects entrenched social inequities [31], institutionalized racism [28], and impacts of discriminatory immigration, employment, and social policy practices [32] experienced by Pacific peoples in NZ.

We also provide some of the first sleep data on Asian school-aged children in NZ, with evidence that Asian children are disproportionately impacted by short sleep (18%) and are less likely to have long sleep durations compared to European/Other children. This inequitable patterning of short sleep compares with a greater proportion of Asian preschoolers [3] and adolescents [33] previously reported to have short sleep compared with European/Other and New Zealand European youth respectively. However, due to a paucity of research, it is unclear whether similar sleep inequities exist for Asian adults in NZ.

Almost one quarter (24%) of Asian children in our study were living in neighborhoods with highest socioeconomic deprivation which was significantly larger than European/Other children. The slope of the distribution of neighborhood deprivation was flatter than that for Māori and Pacific children who were starkly over-­represented in the most deprived areas. In addition, individual-­level deprivation scores did not differ significantly between Asian and European/Other children. Further investigation is warranted to examine the range of social determinants that may contribute to inequities in short sleep experienced by Asian children in NZ and to understand the patterning of short and long sleep for Asian adults.

Our study findings on the sleep of Pacific and Asian children compare with recent research in the US on the sleep of minoritized children which found that in a sample of 9 to 13-year-olds (*n* = 4207) black children slept, on average, 34 minutes less per night than White children [2]. It also aligns with US research findings that racial/ethnic minority children and those living with greatest socioeconomic deprivation are at greater risk of having SDB [34].

One of the strengths of this study design was our ability to identify where ethnic sleep inequities exist, and what some of the drivers of such inequities might be. We found that socioeconomic deprivation was significantly associated with short sleep and symptoms of SDB, independent of ethnicity, age, and gender, and a dose–response relationship was observed whereby increasing neighborhood deprivation was associated with increasingly higher odds of short sleep. Our results add to the growing body of international literature on social determinants of sleep [35] and sleep inequities [36] and provide further evidence that sleep inequities are societally driven and require sociopolitical action to be eliminated.

Our findings support recent international systematic reviews which indicate that low neighborhood socioeconomic status is associated with shorter sleep durations and SDB for children [37, 38]. They also build on our previous research by providing evidence that socioeconomic deprivation is a significant driver of ethnic inequities from early childhood through to adulthood [6]. The use of survey data in this study meant that we were unable to examine mechanisms involved in the independent relationship between area-level socioeconomic deprivation and sleep. However, it is feasible that children living in more deprived neighborhoods were more likely to be living in noisy, cold, damp, poorer quality, and crowded houses [39] with spaces that were less conducive to sleep [40]. They may also have experienced higher levels of stress, such as due to neighborhood safety concerns, which negatively impacted sleep via worries at bedtime [41]. Reasons as to why individual-level deprivation was not independently associated with poor sleep or mental health are less clear, although results were likely impacted by this measure only being available in the 2013–2014 NZHS.

The association between socioeconomic deprivation and poor sleep health in our study appeared to be largely driven by the inequitable distribution of deprivation for Māori and Pacific children and, to a lesser extent, for Asian children. Socioeconomic deprivation did not fully account for associations between ethnicity and sleep indicating that other social determinants were also involved. Exposure to racism may have contributed to relationships between ethnicity and sleep, although we were unable to investigate this due to racism not being included in the NZHS datasets that were analyzed. Direct and indirect (vicarious) experiences of racial discrimination have been associated with poorer sleep health, including shorter sleep durations, and difficulties falling asleep and waking during the night, in school-aged children in Australia [42].

Exposure to racism is more prevalent for Māori, Pacific, and Asian children compared to European/Other children in NZ [16]. This is an area warranting further investigation in relation to school-aged children’s sleep and highlights the importance of including questions on racism as a determinant of health in national child health surveys. This would enable a population-­level investigation of the complex interplay between ethnicity, racism, sleep, and child well-being. The fact that there was a trend in our results for a smaller percentage of Māori and Pacific children to be diagnosed with a sleep disorder yet have significantly higher rates of SDB symptomatology compared to European/Other children also suggests potential inequitable access to clinical sleep services, and racial discrimination and bias within the healthcare system [43]. In NZ, the Ministry of Health has made commitments to equity and anti-racism as part of their responsibilities under Te Tiriti o Waitangi (the Treaty of Waitangi, NZ’s founding document) [44]. Redressing the inequitable distribution of social determinants of health experienced by Indigenous and minoritized children is the first step towards achieving sleep health equity.

We established independent links between short sleep duration and child mental health, including anxiety, ADD/ADHD, and activity-limiting emotional or psychological conditions that impact day-to-day engagement in activities. Thus, we add to evidence on the importance of sufficient sleep duration for children’s waking functioning and mental well-being. We also contribute to the under-studied area of long sleep and child health [20] by providing evidence of an independent relationship between long sleep duration and the higher likelihood of depression in school-aged children. However, as analyses were cross-sectional it is feasible that depressive symptoms may have resulted in children experiencing social withdrawal including spending more time in bed. Thus, further investigation is needed to better understand the links between sleep durations longer than recommended and health outcomes for children.

Snoring and/or breathing noisily during sleep was not significantly associated with any of the mental health measures after adjusting for socioeconomic deprivation, age, and gender. In contrast, previous research has found SDB symptomatology to be associated with a greater likelihood of ADHD and for SDB to be a common comorbid condition with ADHD [45]. Differences in our findings may, in part, be due to the wording of the questions in the NZHS. The question about snoring was not based on diagnostic criteria and could therefore only be used as a proxy indicator of potential SDB. Whereas, the ADD/ADHD question stipulated a formal diagnosis which likely under-represented the number of children with symptoms due to barriers to healthcare utilization [43] and therefore may have contributed to a lack of association. Further investigation in this space is therefore warranted.

Nonetheless, the inequities in short sleep that we identified, and recent research indicating that sleep health is causally related to child mental health whereby improving sleep leads to improved mental health outcomes [46], suggests that sleep inequities may have a “flow on” effect on Māori, Pacific, and Asian children’s mental health. In NZ ethnic inequities exist in child mental health, for example, Māori children are 1.8 times more likely than non-Māori children, and Pacific children are 1.3 times more likely than non-­Pacific children, to experience social, emotional, and behavioral difficulties after adjusting for age and sex [47]. Depressive symptoms are also more prevalent for Māori and East Asian youth and suicide attempts are more prevalent for Māori, Pacific, and South Asian young people, compared to European [48, 49].

This adds further weight to the need for urgent sociopolitical action to tackle social inequities related to sleep inequities and associated poor mental health, and to do so early in the lifecourse to prevent potential entrenched sleep and mental health inequities. Barriers to sleep- and mental health-related healthcare and services must also be addressed to ensure equitable access, sleep assessment, and interventions. Given that we analyzed NZHS data that were collected before COVID-19, this is now of even greater urgency as the pandemic widened social and health inequities and disproportionately impacted Māori and Pacific peoples in NZ [50].

Several limitations of this research must be acknowledged. NZHS data were cross-sectional, therefore we were unable to use formal mediation analysis to investigate causal relationships and recognize that relationships between sleep and mental health may have been bi-directional whereby mental health may also have negatively influenced children’s sleep. All measures were based on primary caregiver report therefore child sleep durations may have differed from child report or objectively measured total sleep time. Sleep duration was the only measure included in multiple surveys resulting in lower numbers of sleep module questions and the inability to investigate other facets of child sleep health, such as sleep quality, timing, and efficiency [51]. As already touched on, the questions on depression, anxiety, and ADD/ADHD likely under-represented the degree of mental distress experienced by children as positive responses were dependent on a formal diagnosis which may have been hindered by inequitable access to health services [43] and biases within the health system. This may therefore have weakened associations between poor sleep and mental health in analyses.

However, a strength of our research was the use of large nationally representative datasets specifically designed to ensure equal explanatory power for Māori, Pacific, and Asian participants. This provided the opportunity to investigate both ethnic inequities in the sleep of 5–14-year-olds and relationships with multiple mental health measures which, to the best of our knowledge, is the first study of its kind in NZ to do so. This was also made possible by the inclusion of consistently worded sleep duration questions in multiple NZHS’s plus the addition of a sleep module in the 2013–2014 NZHS which enabled the investigation of inequities in SDB symptomology at the population level.

In conclusion, sleep inequities exist for Indigenous and minoritized school-aged children in NZ whereby short sleep is more prevalent for Māori, Pacific, and Asian children, and Māori and Pacific children are disproportionately impacted by symptoms of SDB, compared to European/Other children. This is a major public health concern, given that good sleep health is a vital aspect of child well-being in its own right and that our findings established independent associations between short sleep and higher odds of poor mental health, including anxiety, ADD/ADHD, and emotional and psychological difficulties that impact children’s engagement in day-to-day activities. Socioeconomic deprivation was a key, but not sole, driver of these sleep inequities therefore further research is needed to better understand additional social determinants of poor child sleep health such as racism. Action must be taken to tackle sociopolitical drivers of unjust social inequities to prevent entrenched sleep inequities and associated impacts on mental health in childhood and across the lifecourse.

## Supplementary Material

zpad049_suppl_Supplementary_Figures_S1_Tables_S1

## Data Availability

Access to the data used in this study was provided by Statistics New Zealand under conditions designed to keep individual information secure in accordance with requirements of the Statistics Act 1975. The opinions presented are those of the authors and do not necessarily represent an official view of Statistics New Zealand.
