# Supplementary material for: Sleep inequities and associations between poor sleep and mental health for school-aged children: findings from the New Zealand Health Survey
Source: Sleep Adv. 2023 Nov 18;4(1):zpad049. doi: 10.1093/sleepadvances/zpad049 (PMC10710543; doi:10.1093/sleepadvances/zpad049)
Supplement: zpad049_suppl_Supplementary_Figures_S1_Tables_S1 [file zpad049_suppl_supplementary_figures_s1_tables_s1.docx]

Supplementary Material: Table S1 and Figure S1

Title: Sleep inequities and associations between poor sleep and mental health for school-aged children: Findings from the New Zealand Health Survey

Author names and affiliations:

Dr Diane Muller^a*^
Professor T. Leigh Signal^a^

Ms Mathangi Shanthakumar^b^
Associate Professor Sarah-Jane Paine^c^

^a^ Sleep/Wake Research Centre, School of Health Sciences, College of Health, Massey University Wellington, New Zealand

^b^ Environmental Health Intelligence NZ, Massey University Wellington, New Zealand

^c^ Te Kupenga Hauora Māori, Faculty of Medical and Health Sciences, University of Auckland, New Zealand

^*^Corresponding author:

Dr Diane (Dee) Muller
Sleep/Wake Research Centre
School of Health Sciences
College of Health
Massey University
PO Box 756
Wellington 6140
New Zealand
Email: [d.p.muller@massey.ac.nz](mailto:d.p.muller@massey.ac.nz)

Table S1. Logistic regression models (with weighting): Associations between snoring and/or noisy breathing during sleep^a^ and poor mental health^b^ for 5- to 14-year-olds based on 2013/14 New Zealand Health Survey data

|  | Anxiety^c^ | ADD/ADHD^c^ | Activity-limiting emotional problem^d^ | Activity-limiting psychological condition^e^ |
| --- | --- | --- | --- | --- |
|  | OR (95% CI) | OR (95% CI) | OR (95% CI) | OR (95% CI) |
| Unadjusted model | | | | |
| Snoring^a^:  Yes  No | 0.76 (0.36-1.59)  Ref | **2.48 (1.22-5.05)**  Ref | **1.69 (1.13-2.53)**  Ref | **1.92 (1.10-3.34)**  Ref |
| Adjusted model^f^ | | | | |
| Snoring^a^:  Yes  No | 0.69 (0.31-1.55)  Ref | 2.06 (0.93-4.53)  Ref | 1.48 (0.99-2.22)  Ref | 1.64 (0.90-2.98)  Ref |
| Ethnicity^g^:  Māori  Pacific  Asian  European/Other | 1.28 (0.50-3.27)  0.26 (0.05-1.43)  0.97 (0.27-3.49)  Ref | 1.34 (0.41-4.33)  0.28 (0.04-2.18)  **0.08 (0.04-0.16)**  Ref | 0.99 (0.62-1.58)  0.52 (0.25-1.07)  0.66 (0.31-1.43)  Ref | 1.59 (0.74-3.43)  0.48 (0.16-1.40)  1.26 (0.47-3.41)  Ref |
| Age:  5-9  10-14 | Ref  **2.01 (1.13-3.58)** | Ref  1.54 (0.66-3.59) | Ref  1.36 (0.91-2.03) | Ref  1.69 (0.93-3.06) |
| Gender:  Female  Male | **0.38 (0.22-0.67)**  Ref | **0.14 (0.06-0.32)**  Ref | **0.42 (0.27-0.66)**  Ref | **0.32 (0.19-0.55)**  Ref |
| Neighborhood deprivation^h^:  1 (least deprived)  2  3  4  5 (most deprived) | Ref  1.08 (0.39-2.95)  0.70 (0.21-2.38)  0.95 (0.35-2.56)  0.56 (0.15-2.09) | Ref  2.71 (0.51-14.35)  2.59 (0.46-14.56)  1.30 (0.28-6.11)  0.92 (0.17-4.87) | Ref  2.19 (0.91-5.26)  1.52 (0.69-3.34)  1.68 (0.77-3.67)  1.64 (0.68-3.95) | Ref  1.91 (0.53-6.92)  2.11 (0.69-6.47)  2.08 (0.72-6.06)  1.28 (0.38-4.36) |
| Individual-level deprivation^i^:  1 (least deprived)  2  3  4  5 (most deprived) | Ref  0.69 (0.23-2.14)  0.76 (0.27-2.13)  2.36 (0.94-5.95)  2.12 (0.76-5.93) | Ref  2.05 (0.53-7.97)  1.84 (0.38-9.01)  3.54 (0.72-17.33)  3.43 (0.74-15.84) | Ref  1.13 (0.64-2.00)  1.35 (0.79-2.32)  **1.75(1.03-2.98)**  **3.18 (1.86-5.42)** | Ref  1.97 (0.81-4.79)  1.41 (0.52-3.81)  2.50 (0.96-6.51)  2.00 (0.69-5.74) |

*Note.* OR = odds ratio; CI = confidence interval; Ref = reference category.
^a^Based on response to the question “In the last 4 weeks did [child] snore or breathe noisily on most nights, whilst sleeping?”; ^b^Missing outputs for the model investigating associations between snoring and/or noisy breathing during sleep and diagnosed depression due to low counts; ^c^Diagnosed by a health professional; ^d^Based on response to the question “Most children have occasional emotional, nervous, or behavioral problems. Does [child] have any of these problems long-term, that limits the type or amount of activity that [he/she] can do?”; ^e^Based on response to the question “Does a long-term psychological or mental health condition make it difficult for [child] to do everyday activities?”; ^f^Concurrently adjusted for sleep, ethnicity, age, gender, neighborhood deprivation and individual-level deprivation; ^g^Ethnicity in prioritized order of Māori, Pacific, Asian, European/Other (NB: European/Other grouping was based on the format of the data made available to the team by Statistics New Zealand); ^h^Area-level New Zealand Deprivation Index 2013 (NZDep2013) quintiles indicative of relative socioeconomic deprivation based on eight dimensions of deprivation (communication, income, employment, qualifications, home ownership, support, living space, living conditions; ^i^Based on New Zealand Index of Socioeconomic Deprivation for Individuals (NZiDep) scores: 1 = no deprivation characteristics, 2 = 1 deprivation characteristic, 3 = 2 deprivation characteristics, 4 = 3 or 4 deprivation characteristics, 5 = ≥5 deprivation characteristics, based on responses to questions about experiences of deprivation in the past 12 months (buying cheaper food to pay for other things needed; being out of paid work for more than one month; being on a means-tested benefit; feeling cold to save on heating costs; making use of special food grants or food banks; wearing worn-out shoes with holes because cannot afford replacement; going without fresh fruit and vegetables often to pay for other things needed; help from community organizations in the form of clothes or money)

Snoring and/or noisy breathing during sleep

Diagnosed sleep disorders

*Note.* *Denotes statistically significant difference in prevalence compared to European/Other. Ref = reference.

Figure S1. Weighted prevalence estimates of snoring and/or noisy breathing during sleep, and diagnosed sleep disorders, by total Māori, total Pacific, total Asian and sole European/Other ethnicity
